# Freeze-Dried Probiotic Fermented Camel Milk Enriched with Ajwa Date Pulp: Evaluation of Functional Properties, Probiotic Viability, and In Vitro Antidiabetic and Anticancer Activities

**DOI:** 10.3390/foods14152698

**Published:** 2025-07-31

**Authors:** Sally S. Sakr, Hassan Barakat

**Affiliations:** Department of Food Science and Human Nutrition, College of Agriculture and Food, Qassim University, Buraydah 51452, Saudi Arabia; s.sakr@qu.edu.sa

**Keywords:** fermented camel milk, Ajwa dates, probiotics, antidiabetic, anticancer, functional food, food supply

## Abstract

Noncommunicable diseases (NCDs) like diabetes and cancer drive demand for therapeutic functional foods. This study developed freeze-dried fermented camel milk (FCM) with Ajwa date pulp (ADP), evaluating its physical and functional properties, probiotic survival, and potential benefits for diabetes and cancer. To achieve this target, six FCM formulations were prepared using ABT-5 starter culture (containing *Lactobacillus acidophilus*, *Bifidobacterium bifidum*, and *Streptococcus thermophilus*) with or without *Lacticaseibacillus rhamnosus* B-1937 and ADP (12% or 15%). The samples were freeze-dried, and their functional properties, such as water activity, dispersibility, water absorption capacity, water absorption index, water solubility index, insolubility index, and sedimentation, were assessed. Reconstitution properties such as density, flowability, air content, porosity, loose bulk density, packed bulk density, particle density, carrier index, Hausner ratio, porosity, and density were examined. In addition, color and probiotic survivability under simulated gastrointestinal conditions were analyzed. Also, antidiabetic potential was assessed via α-amylase and α-glucosidase inhibition assays, while cytotoxicity was evaluated using the MTT assay on Caco-2 cells. The results show that ADP supplementation significantly improved dispersibility (up to 72.73% in FCM15D+L). These improvements are attributed to changes in particle size distribution and increased carbohydrate and mineral content, which facilitate powder rehydration and reduce clumping. All FCM variants demonstrated low water activity (0.196–0.226), indicating good potential for shelf stability. The reconstitution properties revealed that FCM powders with ADP had higher bulk and packed densities but lower particle density and porosity than controls. Including ADP reduced interstitial air and increased occluded air within the powders, which may minimize oxidation risks and improve packaging efficiency. ADP incorporation resulted in a significant decrease in lightness (L*) and increases in redness (a*) and yellowness (b*), with greater pigment and phenolic content at higher ADP levels. These changes reflect the natural colorants and browning reactions associated with ADP, leading to a more intense and visually distinct product. Probiotic survivability was higher in ADP-fortified samples, with *L. acidophilus* and *B. bifidum* showing resilience in intestinal conditions. The FCM15D+L formulation exhibited potent antidiabetic effects, with IC_50_ values of 111.43 μg mL^−1^ for α-amylase and 77.21 μg mL^−1^ for α-glucosidase activities, though lower than control FCM (8.37 and 10.74 μg mL^−1^, respectively). Cytotoxicity against Caco-2 cells was most potent in non-ADP samples (IC_50_: 82.22 μg mL^−1^ for FCM), suggesting ADP and *L. rhamnosus* may reduce antiproliferative effects due to proteolytic activity. In conclusion, the study demonstrates that ADP-enriched FCM is a promising functional food with enhanced probiotic viability, antidiabetic potential, and desirable physical properties. This work highlights the potential of camel milk and date synergies in combating some NCDs in vitro, suggesting potential for functional food application.

## 1. Introduction

Functional foods are in demand to lower disease risk and improve well-being, especially for noncommunicable diseases (NCDs) like diabetes, heart disease, and cancer. Diets rich in bioactive elements enhance quality of life and reduce chronic illness risk [1]. Camel milk (CM) is rich in proteins, vitamins, and bioactive compounds, providing therapeutic benefits. Unlike bovine milk, it is better tolerated by those with milk allergies and diabetics, making it a promising base for health-promoting dairy products [2,3]. The preservation of these functional components is best achieved through freeze-drying, which is superior to spray drying in maintaining nutrient integrity [4]. Recent studies show fermented camel milk (FCM) helps prevent and manage NCDs like diabetes, hypertension, and inflammation due to its bioactive peptides, antioxidants, and probiotics, supported by preclinical and clinical evidence [5,6]. Notably, FCM selectively targets abnormal and malignant cells with minimal harm to healthy cells, highlighting its therapeutic potential as a functional food [7]. FCM’s probiotics boost gut microbiota diversity, supporting digestive, metabolic, and immune health, while bioactive molecules like lactoferrin and immunoglobulins offer antimicrobial and antiviral benefits [6].

Ajwa dates (*Phoenix dactylifera* L.) are another nutrient-dense food with significant health benefits, containing carbohydrates, dietary fiber, proteins, vitamins, minerals, and a variety of bioactive compounds, such as flavonoids, phenolic acids, and carotenoids [8]. These compounds confer antioxidant and anti-inflammatory properties, demonstrating the inhibition of α-amylase and α-glucosidase [9,10]. In addition, they support heart health, combat oxidative stress, and have antimicrobial properties, but their high natural sugar means intake should be limited, especially for blood sugar management [10,11].

Incorporating Ajwa dates into FCM improves the nutritional profile by increasing total solids, phenolic content, and antioxidant activity, acts as a prebiotic to support beneficial bacteria, and enhances sensory qualities [12]. FCM is particularly well-suited as a probiotic carrier due to its nutraceutical and immunomodulatory properties, offering enhanced health benefits compared to other substrates [13,14]. Camel milk’s unique structure reduces coagulum formation in acidic environments and improves gastrointestinal health, making it an ideal matrix for probiotic delivery [15]. The combination of probiotics and bioactive peptides in FCM contributes to various therapeutic effects, including antihypertensive, antidiabetic, antimicrobial, anti-carcinogenic, and anticholesterolemic activities [5,16]. Fermenting CM with probiotic LAB releases bioactive peptides and metabolites with antioxidant, free radical scavenging, metal chelation, and antimicrobial effects, boosting its functional benefits and supporting gut health and safety [17,18].

Starter cultures in the current study were selected based on their technological performance in fermentation (rapid acidification, flavor, and texture development) and their probiotic potential. *L. acidophilus* LA-5 and *Bifidobacterium bifidum* BB-12 are well-documented commercial probiotics, proven to survive gastrointestinal transit and confer health benefits [19]. *Streptococcus thermophilus* is a traditional dairy starter essential for rapid acidification and flavor, but it is generally not considered a probiotic due to limited evidence of health benefits [20]. *L. rhamnosus* B-1937 may have probiotic potential, as related strains like *L. rhamnosus* GG are established probiotics, but B-1937’s specific properties require confirmation [21]. This leverages *S. thermophilus* for efficient fermentation, while *L. acidophilus* LA-5 and *B. bifidum* BB-12 provide proven probiotic effects. *L. rhamnosus* B-1937 is included for its potential health benefits and to enhance microbial synergy, optimizing both product quality and functional properties. Postbiotics like GABA and bioactive peptides with ACE-inhibitory and antioxidant effects are stronger in FCM, especially when fermented with certain lactic acid bacteria [22,23].

Maintaining probiotic survival during processing and storage is vital for health benefits. Probiotics survive digestion better in camel milk than bovine milk, and additives like skim milk and trehalose improve viability during freeze-drying [24,25]. Bacteria survive stresses through stress protein synthesis, compatible solute accumulation, and cross-protection strategies that enhance resilience during processing [26]. Therefore, optimizing the drying process and storage is essential for maintaining high probiotic cell viability [27].

Combining CM and ADP offers unique functional benefits by modulating the gut microbiome and promoting beneficial bacteria. Freeze-dried probiotic CM with ADP maintains probiotic stability, preserves nutrition, and adds Ajwa’s health benefits. Addressing this gap, the present study aims to develop a novel freeze-dried fermented dairy product combining CM and ADP that is designed to act as an integrated prebiotic-probiotic-postbiotic system. The research hypothesizes that this product can modulate in vitro health-related functions relevant to Type-2 diabetes management and enhance the resistance of Caco-2 cells to cytotoxicity, thereby contributing to noncommunicable disease (NCD) prevention. Additionally, the study evaluates key functional properties of the product, including reconstitution behavior and solubility, to assess its potential applicability in nutritional and functional food sectors.

## 2. Materials and Methods

### 2.1. Materials

Morning raw camel milk was collected from Wadah species camels, aged 5 to 8 years, at the College of Agriculture and Food’s farm at Qassim University in the Qassim province of Saudi Arabia from September to November 2024. The proximate analysis of 100 mL of this raw camel milk indicates it contains 88.9 g of water, 3.04 g of protein, 2.75 g of fat, 8.35 g of non-fat solids, 4.56 g of lactose, and 0.75 g of ash [28]. Al-Madeine Organic Ajwa Dates (*P. dactylifera* L.), harvested in 2024, were sourced from Nakheel Aldka LTD Company in Riyadh, Saudi Arabia. The proximate analysis of 100 g of fresh Ajwa dates, without kernels, shows they contained 18.47 g of water, 2.55 g of protein, 0.32 g of fat, 2.71 g of ash, 75.95 g of total carbohydrates, and 9.15 g of dietary fiber. The Direct Vat Set (DVS) ABT-5 starter culture, obtained from Chr. Hansen Laboratories in Copenhagen, Denmark, includes *L. acidophilus* LA-5, *B. bifidum* BB-12, and *S. thermophilus*. This starter culture was procured from Misr Food Additives (MIFAD) in Badr City, Egypt. Additionally, *L. rhamnosus* (B-1937) was acquired in freeze-dried form as a pure strain from the Agriculture Research Service (ARS) Culture Collection, Norwegian Radio Relay League (NRRL), in Oslo, Norway.

### 2.2. Ajwa Date and Milk Preparation

The Ajwa dates (AADs) were carefully washed, air-dried, hulled, and homogenized to create a consistent ADP. Fresh camel milk, totaling six liters, was transported to the laboratory under refrigerated conditions within one hour post-lactation. The camel milk was divided into three batches, each containing two liters. The first batch was left untreated, the second batch was mixed with 12% ADP (in an 88:12 *v*:*v* ratio), and the third batch was mixed with 15% ADP (in an 85:15 *v*:*v* ratio). All three batches were then heated with agitation until they reached a temperature of 40–50 °C. After standing for 10 min, the mixtures were filtered through two layers of cheesecloth. The filtered milk was sterilized at 75 °C for 5 min, cooled to 40 °C, and then subjected to various treatments with specific cultures, as detailed in Section 2.4.

### 2.3. Propagation of L. rhamnosus B-1937

A pre-activated culture of *L. rhamnosus* B-1937 was aseptically inoculated into sterilized skimmed milk at a concentration of 2% (*v*:*v*). The inoculated milk was then incubated at 40 °C for 24 h for optimal bacterial growth and fermentation. Following incubation, the culture was stored at 4 °C for approximately 18 h to stabilize the cells before being used to prepare fermented camel milk. This process ensured a high viable cell count and consistent probiotic activity in the final product [29].

### 2.4. Preparation of Fermented Probiotic Camel Milk

**Figure 1:**
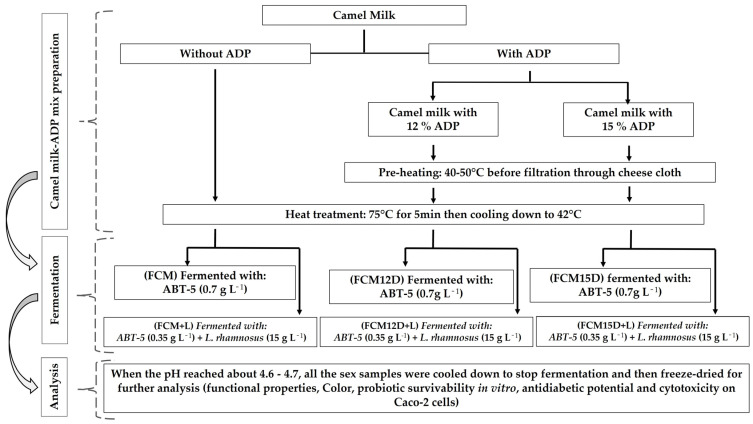
Flow chart for preparing different fermented probiotic camel milk that incorporated ADP fermented with ABT-5 alone or combined with *L. rhamnosus* B-1937.

Six recipes of FCM were developed, utilizing either ABT-5 or a combination of ABT-5 and *L. rhamnosus* for fermentation. The preparation and standardization of inoculum concentrations involved culturing bacterial strains, which were adjusted using the plate counting method [29]. This ensures accurate quantification of viable cells, with inoculum concentrations typically standardized around 10^8^ CFU mL^−1^ [19]. The treatments were then delineated as follows: (FCM) refers to fermented camel milk with ABT-5 (0.7 g L^−1^); (FCM+L) denotes fermented camel milk with ABT-5 (0.35 g L^−1^) plus 15 mL of prepared *L. rhamnosus* culture per liter; (FCM12D) indicates fermented camel milk with ABT-5 (0.7 g L^−1^) containing 12% ADP; (FCM12D+L) signifies fermented camel milk with ABT-5 (0.35 g L^−1^) containing 12% ADP plus 15 mL of prepared *L. rhamnosus* culture per liter; (FCM15D) represents fermented camel milk with ABT-5 (0.7 g L^−1^) containing 15% ADP; and (FCM15D+L) describes fermented camel milk with ABT-5 (0.35 g L^−1^) containing 15% ADP plus 15 mL of prepared *L. rhamnosus* culture per liter. All insulated FCMs were incubated at 42 °C for 6 h or until the pH reached 4.6–4.7. The pH of several samples was assessed using a digital portable pH meter (HANNA HI 8314, Romania). After fermentation, the produced FCMs were stored at 4 ± 1 °C for 24 h. Subsequently, several FCMs were cryopreserved at −80 °C overnight and subsequently subjected to lyophilization for 72 h at −52 °C (CHRIST Alpha 1-2 LD plus, Martin Christ Gefriertrocknungsanlagen GmbH, Osterode am Harz, Germany) and 0.06 mbar. The freeze-dried FCMs were pulverized, aseptically transferred into dark glass flasks, and kept at 5 °C in the dark until suitable examination (Figure 1).

### 2.5. Functional and Physical Properties Analyses

#### 2.5.1. Analysis of Functional Properties of Different Freeze-Dried FCM Powders

Various methods outlined by Malik and Sharma [30] were used to determine the functional properties of different freeze-dried FCMs in terms of water activity (^a^w) using a water activity meter (AquaLab model 3TE, Pullman, WA, USA), dispersibility (%), water absorption capacity (mL g^−1^), water absorption index, water solubility index, insolubility index (g), and sedimentation (%).

#### 2.5.2. Reconstitution Properties of Different FCM Freeze-Dried FCM Powders

The reconstitution properties of freeze-dried FCMs were evaluated in terms of density, flowability, air content, and porosity. The methods employed for determining density were referenced by Deshwal et al. [31]. Specifically, techniques for measuring loose bulk density (LBD, g mL^−1^), packed bulk density (PBD, g mL^−1^), and particle density (PD, g mL^−1^) were detailed [32]. The carrier index (CI, %), Hausner ratio, and porosity (%) were quantified as described [33]. Additionally, the assessment of interstitial and occluded air content and the density of powder solids (g cm^3^) was conducted according to the presented methodologies by Meena et al. [34].

#### 2.5.3. Color Analysis

The color determinations of different FCM incorporating ADP fermented with ABT-5 or ABT-5 with *L. rhamnosus* were performed [35]. The Hunter Lab colorimeter (ColorFlex; Reston, VA, USA) was used. For each sample, 20 mL was poured into a Petri dish (leading to a liquid layer approximately 1 cm high). Triplicate distinct readings of each sample surface were conducted for all analytical determinations. Chroma (C) H°, browning index (BI), and color changes (∆E) were computed relative to the values of the control sample according to Aljutaily et al. [36] using the continuity Equations (1)–(4):

(1) $$C\;=\;(a^{2}\;+\;b^{2}{)}^{0.5}$$

(2) $$H{}^{\circ}=\tan^{-1}(\frac{b}{a})$$

(3) $$\mathrm{BI}\;=\frac{[100\left(\frac{\left(a+1.75L\right)}{\left(5.645L\;+\;a\;-\;3.012b\right)}\right)-0.31]}{0.17}$$

(4) $${∆E}^{*}=\;[{\left(∆L\right)}^{2\;}+{\left(∆a\right)}^{2\;}+{\left(∆b\right)}^{2}{]}^{0.5}$$

### 2.6. Survivability of Different Bacterial Strains in Simulated Gastrointestinal Conditions

The method described by Buahom et al. [37] was applied to determine the survivability of each strain during the simulated gastric digestion with minor modifications. A total of 1 g of each freeze-dried FCM was transferred to a sterilized centrifuge tube containing 9 mL of sterile simulated gastric juice (SGJ). The simulated gastric juice (SGJ) was prepared using phosphate-buffered saline (PBS) at a pH of 2.0. The composition included 125 mM sodium chloride (NaCl), 7 mM potassium chloride (KCl), 45 mM sodium bicarbonate (NaHCO_3_), and 3 g/L of pepsin (3X P). Then, the sterile SGJ, which contained 3000 U/mL of pepsin, was filtered through a 0.2 μm membrane filter before use. Samples were then shaken at 150 rpm for 2 h in a water bath set at 45 °C to ensure the digesta’s temperature reached 38 ± 1 °C inside the 50 mL Falcon tubes during the experiment. After 2 h of incubation in SGJ at 38 ± 1 °C, the samples were centrifuged at 4000 rpm for 5 min at 4 °C, followed by washing with 0.85% (*w*:*v*) standard saline solution before exposure to simulated intestinal juice (SIJ) conditions. The bacterial pellets were then resuspended in 9 mL of sterile SIJ, which consisted of PBS buffer at pH 7.0 with 0.5% (*w*:*v*) bile salt and 2 mg mL^−1^ pancreatin (≥3X P), and then incubated at 38 ± 1 °C and shaken at 150 rpm for 3 h. The viable bacterial count for each strain was determined using selective media appropriate for each probiotic species. The viable counts of *S. thermophilus*, *L. acidophilus*, *Bifidobacterium bifidum*, and *L. rhamnosus* were examined using the standard plate count method. Specifically, M17 agar (pH 6.8 ± 0.2 at 25 °C, from Merck) containing 1% lactose was utilized for *S. thermophilus*, which was then incubated at 37 °C for 48 h under aerobic conditions. *L. acidophilus* was counted using MRS agar containing 0.2% OX-gall, with incubation occurring at 37 °C for 72 h in anaerobic jars using a GasPak system (GasPak System-Oxoid, Basingstoke, Hampshire, England), following standard methods. *Bifidobacterium bifidum* was selectively enumerated on MRS agar supplemented with 0.05% L-Cystine hydrochloride, incubated at 37 °C for 72 h under anaerobic conditions. Identification of *L. rhamnosus* was achieved by counting colonies on MRS agar containing 0.05 mg/L vancomycin, with a 72 h incubation period at 40 °C under anaerobic conditions [38,39,40]. The survivability of each strain exposed to the SGJ and SIJ environments was calculated and expressed as the survival % for each digestion stage relative to the initial counts.

### 2.7. Bio-Functionality of Probiotic-Camel Milk Supplemented with ADP

The water-soluble extract (WSE) was initially prepared to assess the bio-functionality of freeze-dried FCM samples, following the methodology outlined by Shori et al. [41]. Each freeze-dried FCM sample without any extra treatment was mixed with 40 mL distilled water, vortexed, and then held at 40 °C for 1 h. before centrifugation (10,000× *g*/30 min/room temperature). After centrifugation, the WSE-clear supernatant was harvested and kept at −80 °C for subsequent analysis.

#### 2.7.1. Antidiabetic Potential

The α-amylase inhibitory activity of the FCM was evaluated using the 3,5-dinitrosalicylic acid (DNSA) colorimetric method of Wickramaratne et al. [42] with minor modifications. The WSE was initially dissolved in a small volume of 10% dimethyl sulfoxide (DMSO) and further diluted with phosphate buffer (0.02 M Na_2_HPO_4_/NaH_2_PO_4_ and 0.006 M NaCl, pH 6.9) to achieve concentrations ranging from 1.9 to 1000 μg mL^−1^. For each reaction, 200 μL of α-amylase solution (2 U mL^−1^; Sigma-Aldrich, St. Louis, MO, USA) was mixed with 200 μL of the extract and incubated at 30 °C for 10 min. Subsequently, 200 μL of a 1% (*w*:*v*) soluble starch solution was added to initiate the reaction, followed by a 3-min incubation. The reaction was terminated by adding 200 μL of DNSA reagent (prepared by dissolving 12 g of sodium potassium tartrate tetrahydrate in 8.0 mL of 2 M NaOH and 20 mL of 96 mM DNSA solution). The reaction mixture was then heated in a water bath at 85–90 °C for 10 min, cooled to room temperature, and diluted with 5 mL of distilled water. The absorbance was measured at 540 nm using a UV-visible spectrophotometer (Biosystem 310, BioSystems S.A., Barcelona, Spain). A control sample representing 100% enzyme activity was prepared by substituting the extract with 200 μL of buffer, and Acarbose α-amylase inhibitor was used as a positive control. Blank samples containing the extract without enzyme were used to correct for background absorbance. The percentage inhibition of α-amylase activity was calculated as follows:

% α amylase inhibition = 100 × (Abs_100%_ control − Abs_extract_)/Abs_100%_ control

The IC_50_ value, representing the concentration required to inhibit 50% of the enzyme activity, was determined from a dose–response curve plotting percent inhibition against extract concentration.

The α-glucosidase inhibitory activity of each FCM sample was assessed according to the method described by Pistia-Brueggeman and Hollingsworth [43], with slight modifications. In this assay, 50 μL of WSE (1.97–1000 μg mL^−1^) was incubated with 10 μL of α-glucosidase solution (1 U mL^−1^; Sigma-Aldrich, St. Louis, MO, USA) and 125 μL of 0.1 M phosphate buffer (pH 6.8) at 37 °C for 20 min. The enzymatic reaction was initiated by adding 20 μL of 1 M p-nitrophenyl-α-D-glucopyranoside (pNPG) and continued for 30 min. The reaction was terminated by adding 50 μL of 0.1 N sodium carbonate (Na_2_CO_3_), and the absorbance was measured at 405 nm using a Biosystem 310 Plus spectrophotometer. One unit of α-glucosidase activity was defined as the amount of enzyme required to release 1 μmol of *p*-nitrophenol from pNPG per minute under the assay conditions. The following equation was used for the α-glucosidase inhibitory activity calculation:

% α-glucosidase inhibitory = 100 × (OD_blank_ − OD_extract_)/OD_blank_

Percentage inhibition was calculated, and the IC_50_ value was determined by plotting the percent inhibition against extract concentration and applying regression analysis.

#### 2.7.2. Anticancer Potential

The cytotoxic potential of FCM extracts was assessed against human colon adenocarcinoma (Caco-2) cell lines using the 3-(4,5-dimethylthiazol-2-yl)-2,5-diphenyltetrazolium bromide (MTT) assay, as described by Sharma et al. [44] with slight modifications. Caco-2 cells were seeded at a density of 1 × 10^5^ cells mL^−1^ into 96-well plates and incubated at 37 °C for 24 h to allow cell adherence and monolayer formation. Following incubation, the medium was discarded, and the wells were gently washed twice with phosphate-buffered saline (PBS). Cells were treated separately with different FCM extracts at 31.25, 62.5, 125, 250, 500, and 1000 μg mL^−1^ concentrations. The plates were incubated at 37 °C for the designated exposure period. Following treatment, 20 μL of MTT solution (5 mg/mL) was added to each well, and the plates were shaken at 150 rpm for 5 min before being incubated at 37 °C for an additional 4 h to facilitate the formation of formazan crystals. After incubation, the medium was carefully removed, and the resulting formazan crystals were solubilized by adding 200 μL of dimethyl sulfoxide (DMSO) to each well. The optical density (OD) was measured at 560 nm with background subtraction at 620 nm using a microplate ELISA reader. The OD values were considered directly proportional to the number of viable cells, allowing for the quantification of cytotoxic effects at each extract concentration.

### 2.8. Statistical Analysis

Data are expressed as the mean ± standard error (SE). A randomized complete block design and analysis of variance of factorial methods were carried out using the SPSS program (SPSS Ver. 23, IBM, Armonk, NY, USA). All data were analyzed in three replications for each parameter, following Steel [45]. Tukey’s multiple-range test was used to conduct the one-way ANOVA for experimental design and comparisons, and the results were considered statistically significant at *p* ≤ 0.05.

## 3. Results and Discussion

### 3.1. Functional Properties of Different FCM Powders

**Figure 2:**
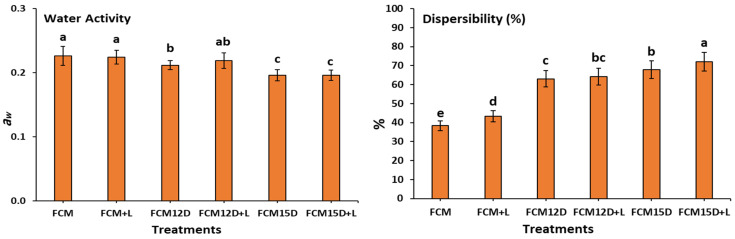

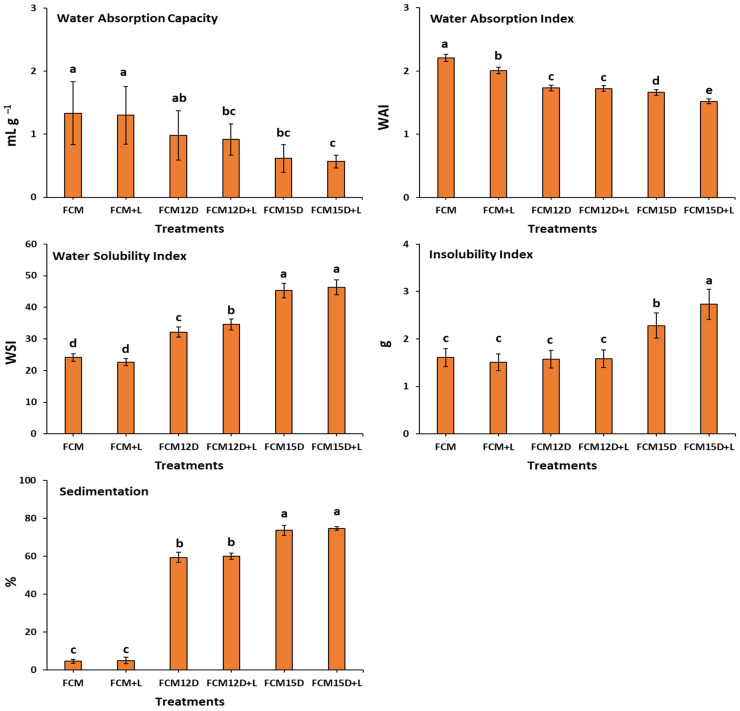
The functional properties of freeze-dried FCM and FCM incorporating ADP fermented by either ATB-5 or ATB-5 with *L. rhamnosus* for 6 h. Treatments: (FCM, fermented camel milk with ABT-5 (0.7 g L^−1^); FCM+L, fermented camel milk with ABT-5 (0.35 g L^−1^) + 15 mL of *L. rhamnosus*; FCM12D, fermented camel milk with ABT-5 (0.7 g L^−1^) incorporating 12% ADP; FCM12D+L, fermented camel milk with ABT-5 (0.35 g L^−1^) incorporating 12% ADP + 15 mL of *L. rhamnosus*; FCM15D, fermented camel milk with ABT-5 (0.7 g L^−1^) incorporating 15% ADP; and FCM15D+L, fermented camel milk with ABT-5 (0.35 g L^−1^) incorporating 15% ADP + 15 mL of *L. rhamnosus* culture). ^a,b,c,d,e^: Bars sharing the same letters are not statistically different (*p* ≤ 0.05).

Figure 2 presents the functional properties of FCMs. The dispersibility of freeze-dried FCMs was the highest in samples supplemented with either 15% or 12% ADP. It was recorded at 72.73% for FCM15D+L, followed by 67.85, 64.20, and 63.05% for FCM15D, FCM12D+L, and FCM12D samples. The lowest solubility was for the samples without ADP supplementation (FCM+L: 43.32 and FCM: 38.31). Our results parallel those of How et al. [46], indicating that powders with high bulk densities (Table 1) exhibit better dispersibility (Figure 2), as they can penetrate more easily than those with low bulk densities. Notably, adding ADP during FCM formulation significantly improved the dispersibility of freeze-dried FCM powder (Figure 2). Dispersibility is a critical technological property influencing the rehydration and solubilization efficiency of freeze-dried powders, as it reflects the powder’s ability to separate and resist clumping, which is essential for consistent texture and bioavailability in functional foods [46]. The observed enhancement in dispersibility upon ADP addition likely results from changes in powder morphology and particle interactions, facilitating better water penetration and particle separation [46]. Studies have indicated that dispersibility decreases as particle size decreases, with smaller particles often forming visible lumps that further reduce dispersibility [47].

Thus, enhancing the dispersibility of the FCM samples is associated with the increased addition of ADP, which may arrange the particle sizes of various powder samples [48,49]. Regarding the above, increasing the FCM sample’s dispersibility is associated with increasing the amount of ADP added, which may be a reason for rearranging the particle size in different powder samples. The water activity, water absorption capacity, and water absorption index of the freeze-dried powder from FCMs ranged from 0.196 to 0.226, 0.567 to 1.33, and 1.52 to 2.21, respectively. The amount of ADP added significantly influenced these measurements. During the freeze-drying process, water is eliminated through the sublimation of ice, resulting in a porous matrix that enhances capillary forces while reducing the water activity of the sample [50]. The disparity in our findings may be attributed to the initial moisture content of the samples before freeze-drying. Lower moisture content yields a corresponding decrease in porosity percentage (Table 1) for the same sample. This study’s findings indicate that all freeze-dried food contact materials (FCMs) exhibit low water activity levels, aligning with the recommended range of 0.20–0.40 for dried foods, as previously mentioned [51]. This observation suggests that these samples could possess stability characteristics that make them viable candidates for use as long-term supplements.

Solubility represents the concluding phase of reconstitution, wherein powder particles disaggregate into individual entities. The water solubility index quantifies the extent of soluble degradation in carbohydrates and other disposable substances [51,52]. The observed elevation in carbohydrates and minerals, alongside a reduction in protein and fat, in samples with the addition of ADP may contribute to the increased water solubility index (Figure 2) in these samples compared to others. The highest insolubility index (FCM15D+L: 2.73 g) and sedimentation rate (FCM15D+L: 75.67%) were identified in samples enriched with elevated levels of ADP. In contrast, the control samples, which lacked ADP, demonstrated significantly lower values for both metrics. The insolubility index for FCM was measured at 1.61 g, while FCM+L exhibited an index of 1.51 g. Correspondingly, the sedimentation rates for these samples were recorded at 4.67% and 5.00%, respectively. The results of Malik and Sharma et al. [30] were in line with ours. They attributed these results to solubility playing a critical role in the behavior of powdered substances, as lower solubility indices can result in problematic sedimentation during reconstitution. Typically, a reduction in particle size enhances the solubility of the powder. Moreover, the inverse relationship between particle size and dispersibility, where smaller particles tend to aggregate and reduce dispersibility, underscores the importance of optimizing particle size distribution during powder formulation to maximize functional performance [47]. Therefore, the improved dispersibility of ADP-supplemented FCM powders suggests enhanced practical applicability in nutritional and functional food products, where rapid and uniform reconstitution is desired for consumer acceptability and efficacy [53].

### 3.2. Reconstitution Properties of Different FCM Powders

The reconstitution or rehydration properties of freeze-dried FCMs are summarized in Table 1. Reconstitution is the process by which powdered substances dissolve in liquid, and it involves several steps: wetting, sinking, dispersing, and dissolving. Rehydrating dairy powders is crucial for utilizing their functional properties, such as gelation and surface activity, and the nutritional bioavailability of their nutritional substances, composed of essential amino acids and bioactive compounds. Adequate rehydration requires uniform wetting, proper dispersion, and thorough solubilization of the powder particles, which is influenced by immiscibility, dispersibility, solubility, composition, particle size, shape, density, and any added substances of the powder [54].

The densities (g mL^−1^) of different freeze-dried FCMs varied significantly, ranging from 0.22 to 0.36 for low bulk density (LBD), 1.24 to 1.44 for low protein bulk density (PBD), 1.61 to 3.03 for powder density (PD), and 1.31 to 1.38 for solid powder densities. Significant differences (*p* ≤ 0.05) in densities were observed among various samples. The highest values for LBD, PBD, and powder solid density, showing no significant differences, were recorded for samples containing ADP (FCM12D, FCM12D+L, FCM15D, and FCM15D+L) compared to those without ADP (FCM and FCM+L). Conversely, a significant increase in particle density (PD) was observed for the FCM and FCM+L samples that were free of ADP compared to the other FCMs. In contrast, a significant reduction in porosity as the amount of ADP addition increased can also be observed between FCMs (Table 1). Those decrements are related to the decrease in the PD of the samples.

The increase in bulk density in dried FCMs was parallel to the increase in SNF% and decrease in fat%, as presented in previous work [28]. This finding is comparable to the observation made by Deshwal et al. [31], who noted that bulk density is increased by high dry matter and skimming. According to How et al. [46], higher bulk densities in powders are often desirable because they enable more powder to be packed into a smaller package. Our results appear promising, particularly in terms of minimizing air space in the packaging. This reduction can help prevent oxidation, as evidenced by the significant decrease in interstitial air content and the increase in occluded air in samples supplemented with higher amounts of ADP. The size of particles significantly impacts the bulk properties of materials. Also, there is an inverse relationship between the size of smooth, spherical particles and the amount of air they contain, which affects bulk density [30].

The significant reduction in porosity% as the amount of date paste addition increased can also be observed between FCMs (Table 1). Those decrements are related to the decrease in the samples’ particle density (PD). Regarding the flow behavior reflected by CI and HR, all FCMs revealed poor flowability. As previously mentioned [30,54], cohesive powders, such as dairy powders, frequently exhibit poor flow characteristics and tend to clump together when exposed to moisture. Indeed, when large agglomerates are in contact with liquid, only the outer surfaces become wet initially, forming a gel layer that hinders further water penetration. This presents substantial challenges for adequately rehydrating freeze-dried fermented milk powders, which could be overcome by adding protectants like maltodextrin and trehalose, as reported by How et al. [46].

Practically, the ability to efficiently reconstitute freeze-dried camel milk powder is critical for its application in functional foods and nutraceuticals. Rapid and uniform rehydration ensures the consistent delivery of probiotics, prebiotics, and bioactive compounds, essential for health benefits such as improved glycemic control and gut health [55,56]. Moreover, the powder form facilitates extended shelf life, easier transportation, and incorporation into diverse food matrices, broadening consumer access to camel milk’s unique nutritional profile [31].

### 3.3. Color Parameters of FCM

The results of the color measurement of FCM incorporating ADP are presented in Table 2. Adding ADP to CM changed the visual color parameters. As previously reported, red, green, blue, and yellow are the colors defined by hue (h); lightness (L*) is the parameter for color brightness; and chromaticity (C), or colorfulness, represents the color sensation, and they are all measured using instrumental colorimeters. Incorporating ADP as a natural sweetener to FCM positively affected their color (Table 2). A significant decrease in lightning (L*) was observed in all samples containing ADP compared to FCM or FCM+L. A significant (*p* ≤ 0.05) increase was also observed in the redness (a*) and yellowness (b*) in all samples that incorporated ADP. The relation between redness (a*) and yellowness (b*) was inverse for all samples with ADP levels. It was observed that the color of FCM fortified with ADP became dim as the content of ADP was gradually increased from 12 to 15%. The value of L* (as an indicator for light vs. dark) decreased significantly (*p* ≤ 0.05) with increasing content of ADP. The mean a* values (as an indicator for red vs. green) were significantly affected by increasing ADP levels.

Furthermore, when the ADP level was increased, FCM enriched with ADP exhibited a higher significance b* value (as an indicator for yellow vs. blue). The H° presented a significant difference between FCM and all FCM-ADP treatments, whereas a non-significant difference was recognized. Most importantly, C, BI, and ∆E increased by increasing ADP levels compared with plain FCM. Visual assessment plays a vital role in evaluating the quality of milk. Measuring the color of FCM and FCM-SKD, we observed a significant change in a positive relationship with increasing ADP levels. This may be due to increased pigments and phenolic compounds as ADP levels increased. Hachani et al. [57] stated that colors primarily result from pigments produced through the condensation of phenolic compounds or browning reactions during ripening and storage.

Increasing H°, BI, and ∆E with increasing ADP levels reflects the rise in phenolic and pigment contents in formulated FCM. However, Gross et al. [58] identified several pigments in dates, including carotenoids (lycopene, carotenes, flavoxanthin, and lutein), anthocyanins, flavones, and flavonols. At the same time, colors are caused by pigments produced by different enzymatic and nonenzymatic reactions. Such pigments include melanoidin and caramels that result from the Maillard reaction and enzymatic reactions catalyzed by polyphenol oxidase (e.g., melanins) or during milk pasteurization [58,59].

Interestingly, increasing ADP levels causes a noticeable darkening (decrease in lightness, L*) and enhanced redness (a*) and yellowness (b*), reflecting higher pigment and phenolic compound content. These visual changes, driven by natural pigments such as carotenoids, anthocyanins, and flavonoids, as well as browning products like melanoidins from Maillard and enzymatic reactions, not only influence consumer perception and acceptance by imparting a richer, more appealing hue but also serve as indirect indicators of bioactive compound stability. The intensified color correlates with increased phenolic content, which is known for antioxidant activity, suggesting that the darker color may better preserve these health-promoting compounds during processing and storage. Thus, the color modifications linked to ADP addition have dual significance: they enhance market appeal through improved visual quality while signaling bioactive constituents’ retention and potential efficacy critical for the product’s functional benefits [60].

### 3.4. Survivability of Different Bacterial Strains in Simulated Gastrointestinal Environments

**Figure 3:**
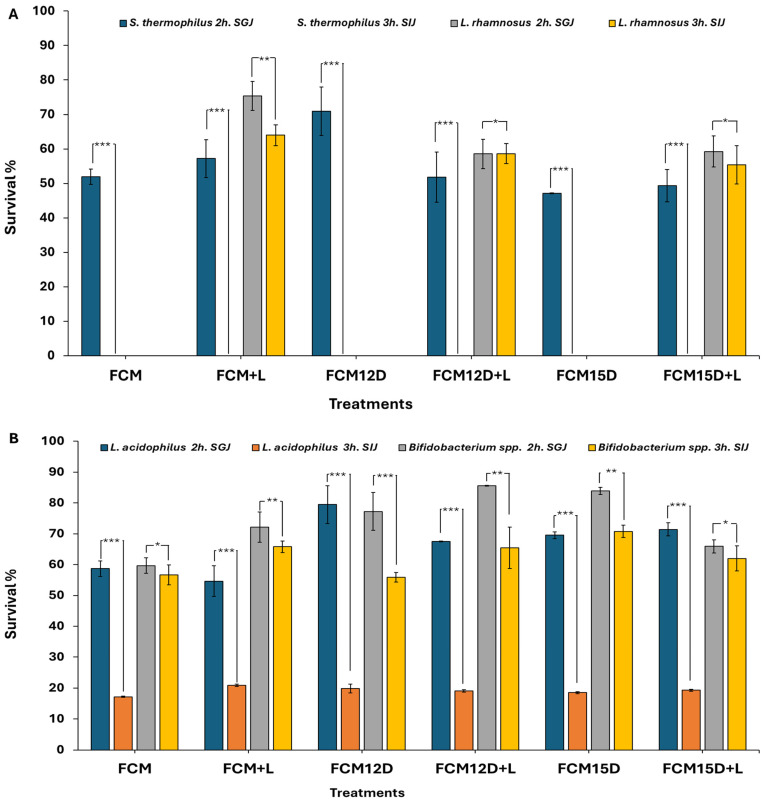
Survival% of different bacterial strains in freeze-dried FCMs, either ATB-5 or ATB-5 with *L. rhamnosus*, for 6 h. (**A**) Survival% of *S. thermophilus* and *L. rhamnosus* under in vitro SGJ (pH 2.0) and SIJ (0.5% bile salt) conditions and (**B**) *L. acidophilus* LA-5 and *B. bifidum* BB-12 under in vitro SGJ (pH 2.0) and SIJ (0.5% bile salt) conditions. Treatments: (FCM, fermented camel milk with ABT-5 (0.7 g L^−1^); FCM+L, fermented camel milk with ABT-5 (0.35 g L^−1^) + 15 mL of *L. rhamnosus*; FCM12D, fermented camel milk with ABT-5 (0.7 g L^−1^) incorporating 12% ADP; FCM12D+L, fermented camel milk with ABT-5 (0.35 g L^−1^) incorporating 12% ADP + 15 mL of *L. rhamnosus*; FCM15D, fermented camel milk with ABT-5 (0.7 g L^−1^) incorporating 15% ADP; and FCM15D+L, fermented camel milk with ABT-5 (0.35 g L^−1^) incorporating 15% ADP + 15 mL of *L. rhamnosus* culture,). *, **, and *** indicate significance at *p* < 0.05, *p* < 0.01, and *p* < 0.001, respectively.

It is essential to establish the health benefits of probiotics and confirm their ability to colonize and proliferate in the human gastrointestinal tract successfully [61]. Therefore, the survival rates of various bacterial strains in freeze-dried FCM samples were monitored during simulated gastric and intestinal phases. Data presented in Figure 3 suggest that the behavior of each strain demonstrates considerable variation following exposure to a simulated gastric environment in vitro, particularly during the intestinal phase. The survival rate of *L. acidophilus*, *B. bifidum*, and *L. rhamnosus* was significantly reduced in the simulated gastric phase compared to the simulated intestinal phase. Conversely, no viability was found for *S. thermophilus* in the simulated intestinal phase. Population reduction during the stomach and intestinal phases is due to stress from pH changes. The stomach’s low pH damages microbial cells, which can be rebuilt in the intestinal phase [62]. As previously mentioned, food components, especially fat and protein, serve as protective factors, creating a buffered environment for the bacteria [63], highlighting the food matrix’s protective role, especially fermented milk, on probiotic cells during gastric conditions [13]. As a limitation, freeze-drying is optimal for nutrient preservation, but it is less efficient than spray-drying in terms of processing time and cost. However, integrating ADP enhances the nutritional value, sensory appeal, and probiotic viability, making it a valuable addition to the formulation.

It is crucial to verify the health benefits of probiotics by confirming their survival and colonization in the human gastrointestinal tract. In this study, freeze-dried FCM showed strain-dependent survival during simulated gastric and intestinal phases, with *L. acidophilus*, *B. bifidum*, and *L. rhamnosus* exhibiting significant viability reductions in the gastric phase but better survival in the intestinal phase. At the same time, *S. thermophilus* failed to survive the intestinal simulation. These findings align with previous research demonstrating that low gastric pH causes microbial stress and viability loss, but the intestinal environment allows partial recovery [64,65]. The protective role of the fermented milk matrix, rich in fat and protein, buffers probiotics against acidic gastric conditions, enhancing their survival, as supported by studies on camel milk probiotic products [64,66]. Significantly, adding ADP improves nutritional value, sensory characteristics, and notably enhances probiotic viability, likely due to its prebiotic components and phenolic compounds that synergistically protect probiotics during digestion [66,67]. These results suggest that incorporating ADP into FCM formulations can improve the functional efficacy and consumer acceptance of probiotic dairy products, supporting their commercial potential in the functional food market.

Indeed, co-culturing of *L. rhamnosus* and *L. acidophilus* on nonselective MRS agar prevents reliable strain discrimination, since both species form virtually identical colonies. Without supplementary methods—such as microscopic examination of cell morphology or molecular assays (e.g., species-specific PCR or 16S rRNA sequencing)—accurate enumeration and assignment of each strain’s contributions will not be accurately counted. This limitation compromises the precision, validity, and reproducibility of the study’s findings and must be explicitly acknowledged in the interpretation of our results.

### 3.5. Antidiabetic and Anticancer Bio-Functionality of FCM

#### 3.5.1. Antidiabetic Properties

Research has indicated that α-amylase and α-glucosidase are crucial enzymes in carbohydrate metabolism. Inhibiting these enzymes can slow down carbohydrate degradation, decreasing blood glucose levels [68]. Studies have shown that fermented camel milk significantly inhibits these carbohydrate-hydrolyzing enzymes. The inhibition rates reported are up to 80.75% for α-amylase and 85.37% for α-glucosidase [69,70]. These findings suggest that fermented camel milk has considerable potential to reduce glucose release from dietary carbohydrates, which may improve glycemic control [71].

As shown in Table 3, adding ADP and including probiotic starter culture strains exerted a statistically significant (*p* ≤ 0.05) positive effect on inhibiting both enzymes. The difference in IC_50_ values of α-amylase and α-glucosidase was significantly decreased by about 32.10 and 17.16 μg mL^−1^, respectively, for the FCM sample compared to the FCM+L sample, indicating a pronounced effect of *L. rhamnosus* in promoting the release of bioactive compounds during fermentation. Furthermore, FCM samples fortified with 12% and 15% ADP and fermented with ABT-5 and *L. rhamnosus* exhibited the least potent in vitro antidiabetic activity among all formulations. Notably, the FCM15D+L sample achieved IC_50_ values of 111.43 μg mL^−1^ for α-amylase and 77.21 μg mL^−1^ for α-glucosidase, highlighting the total reduction in enzyme inhibitory potential found in the FCM extract. Thus, by inhibiting α-amylase and α-glucosidase, fermented milk with *L. rhamnosus* may help slow carbohydrate digestion and glucose absorption, supporting blood sugar regulation [72,73,74]. Along with our results, it has been reported that *L. rhamnosus* strains exhibit substantial α-glucosidase inhibitory activity. For instance, *L. rhamnosus* BD2, isolated from kefir grains, demonstrated an inhibition rate of up to 73.58%; however, this activity declined to 25.72% in milk-based cultures, likely due to alterations in peptide composition during milk fermentation [72]. Similarly, *L. rhamnosus* LB1lac10 has the highest α-glucosidase inhibition among the tested strains, a property attributed to its production of a specific exopolysaccharide, EPS1-1, known for its potent enzyme-inhibitory capabilities [69,75]. Although direct evidence regarding the α-amylase inhibitory potential of *L. rhamnosus* in milk is currently limited, related *Lactobacillus* species present in fermented dairy products have shown α-amylase inhibition ranging from 18.79% to 63.42% [73]. Moreover, fermented camel and bovine milk inoculated with various *Lactobacillus* spp. have exhibited α-amylase inhibition levels exceeding 34% [69,75].

Particularly when FCM is fortified with ADP and fermented using a combination of *L. rhamnosus* and ABT-5, it exhibits less significant inhibitory activity against the carbohydrate-hydrolyzing enzymes α-amylase and α-glucosidase. These results support the potential of FCM as a functional food with antidiabetic properties. Supporting our findings, Çakmakoğlu et al. [76] noted that determining antidiabetic inhibition activities in water-soluble extract exhibited less effect when compared to the low-molecular-weight peptide fractions of the same samples. The significant reduction in IC_50_ for enzyme inhibition observed in ADP-fortified samples (especially FCM15D+L) could be a result of applying the analysis in crude FCM extract without extra peptide fractionations, indicating the importance of the types and concentration of bioactive peptides in the tested sample.

In the same respect, Castañeda-Pérez et al. [77] declared that the most excellent α-glucosidase inhibition (97.34%) was noted in WSEs containing inhibitory peptides >10 kDa. They attributed the presence of hydrophobic amino acids (AAs) in the samples to inhibition. Siow and Gan [78] identified three high molecular weight peptides (17–23 AAs in length) that inhibited α-amylase. Ibrahim et al. [79] reported that amino acid residues with alkali or OH groups on the *n*-terminal side and Pro, Ala, and Met on the C-terminal side inhibit α-glucosidase. Regarding α-amylase inhibitory peptides, Ngoh and Gan [80] confirmed the significance of Leu or Phe at the C-terminal and Phe or Gly at the N-terminal of these peptides to be affected.

The observed inhibitory effects could be attributed to the production of low-molecular-weight peptides (≤3 kDa) and exopolysaccharides generated during fermentation [81]. These bioactive compounds, often originating from the proteolytic breakdown of milk caseins, contribute to enzyme inhibition and antioxidant activity [72,74]. The enhanced proteolytic activity associated with *L. rhamnosus* during fermentation facilitates increased release of these peptides, thereby amplifying the functional properties of the fermented product, including its antioxidant potential and enzyme-inhibitory efficacy [82,83].

These findings are further supported by previously proposed mechanisms underlying fermented dairy products’ in vitro antidiabetic effects [69,70,75]. The inhibitory activity of *Lactobacillus* strains on α-amylase and α-glucosidase largely depends on the type, sequence, and structure of peptides produced during fermentation and their interactions with the active sites of the enzymes. Short-chain peptides show the highest inhibitory efficiency, likely due to their structure and specific amino acid residues that form stable enzyme interactions [84]. A few studies have isolated the bioactive peptides in freeze-dried camel milk responsible for its antidiabetic effects, followed by mass spectrometry, and identified peptides inhibiting enzymes such as α-glucosidase and DPP-IV [85,86].

#### 3.5.2. Cytotoxicity Effect

Many previous studies indicated that fermented camel milk, particularly when enriched with probiotics, exhibits notable cytotoxic effects against Caco-2 cancer cells [69,75]. These effects surpass those observed with fermented bovine milk, likely due to enhanced proteolytic activity and the generation of bioactive compounds during fermentation. However, this highly depends on the probiotic strains and bioactive peptides released during fermentation [44,76]. In the present study, data in Table 3 indicate that fermented camel milk samples exhibited varying degrees of cytotoxicity against the Caco-2 cell line, as reflected by their IC_50_ values. The most substantial cytotoxic effects were observed in samples fermented with ABT-5 alone rather than those fermented with ABT-5 in combination with *L.rhamnosus*. Specifically, the lowest IC_50_ values were recorded for FCM (82.22 μg/mL), followed by FCM+L (85.19 μg mL^−1^) and FCM12D (94.89 μg mL^−1^), indicating higher antiproliferative activity.

In contrast, treatments with extracts from FCM15D, FCM12D+L, and FCM15D+L resulted in less inhibition of Caco-2 cell proliferation, suggesting a weaker cytotoxic response. These results may be linked to differences in proteolysis among the FCM samples, as detailed in our recently published study [28], where an inverse relationship between proteolytic activity and cytotoxicity was observed. This suggests that increased fortification with ADP and incorporating *L.rhamnosus*, a highly proteolytic probiotic strain, during fermentation may reduce the anticancer efficacy of camel milk against Caco-2 cells.

Along with our results, the findings of [69,75] revealed that the fermentation of camel milk with different probiotic strains resulted in different antiproliferation activities against Caco-2 cells. They reported that fermented camel milk, particularly when produced using specific probiotic strains, demonstrates more substantial antiproliferative effects than bovine milk. These effects appear strain-dependent, likely due to the unique peptides generated during fermentation by different bacterial strains. One hypothesis attributes this activity to the specific cytotoxicity of the released peptides, which can trigger apoptosis in cancer cells. Another hypothesis suggests that the peptides interfere with cancer progression by competitively binding to cell membrane receptors, thereby blocking the action of cancer-promoting growth factors. The high antiproliferative activity observed in FCM extracts may thus result from a combination of these mechanisms, including enhanced receptor competition and apoptosis induction. Additionally, the correlation between antiproliferative effects and antioxidant activity suggests these peptides may possess multifunctional bioactivity. Similarly, ref. [87] assessed the antiproliferative activity of probiotic whey beverages fermented with *L. acidophilus* La-05, *L. acidophilus* La-03, *L. casei*-01, and *B. animalis* Bb-12 against the human prostate cancer cell lines DU-145 and PC-3. Their results demonstrated that all tested beverages exerted cytotoxic effects on both cell lines, with the formulation fermented by *L. casei*-01 exhibiting the most significant inhibitory effect, indicating its potential as a promising candidate for prostate cancer intervention. Although the fermentation of camel milk using proteolytic strains such as *L. plantarum* KGL3A [88] and *L. rhamnosus* MTCC 5945 (NS4) has been shown to enhance its functional properties, including increased production of bioactive peptides with antioxidative, antidiabetic, and antihypertensive activities, these processes also significantly improve the sensory attributes and probiotic potential of the resulting product [89]. Solanki and Hati [89]’s experiment resulted in the release of bioactive peptides with molecular weights of 3 kDa, 5 kDa, and 10 kDa, exhibiting enhanced antioxidant and ACE-inhibitory activities. Peptides with molecular weights ≤5 kDa isolated after camel milk fermentation with a mixed culture containing *L. rhamnosus* have demonstrated superior cytotoxic effects [76]. However, under our research conditions, these low-molecular-weight peptides may be present only in small amounts within the total fermented camel milk extract, potentially explaining the lack of significant antiproliferative effects against Caco-2 cells in samples fermented with a mixed starter culture containing *L. rhamnosus*. Therefore, further experimental research is needed to investigate the cytotoxic potential of bioactive peptides of different molecular weights released during camel milk fermentation as prospective anticancer agents.

The observed reduction in antiproliferative effects with higher ADP content underscores the complexity of bioactive peptide interactions within fermented camel milk (FCM) matrices. It highlights the critical need for detailed fractionation and characterization of these peptides to elucidate their mechanisms of action. Recent studies emphasize that bioactive peptides derived from food proteins exhibit diverse anticancer activities—including apoptosis induction, inhibition of cell proliferation, and suppression of metastasis—which are largely dependent on their molecular size, sequence, and structural properties, and can be altered by fermentation conditions and added ingredients like ADP [90,91]. Fractionation techniques such as ultrafiltration and chromatography enable the isolation of specific peptide fractions, precisely identifying those with potent cytotoxicity and clarifying how ADP influences peptide profiles and bioactivity [91,92]. Moreover, advances in peptide-based therapeutics demonstrate that modifications and delivery strategies can enhance stability and selectivity. This suggests that understanding the peptide composition in FCM-ADP is essential for optimizing anticancer potential [93]. However, a key limitation of our study is the absence of comparative analyses using standard anticancer agents alongside the cytotoxicity assessments of the tested compounds. Although we evaluated IC_50_ values to estimate cytotoxic effects without including established chemotherapy drugs as positive controls, it is challenging to contextualize the potency and selectivity of our agents relative to clinically relevant treatments [94,95]. Moreover, vehicle controls and thorough characterization of the Caco-2 cell model, including differentiation status and viability under experimental conditions, were not comprehensively addressed. These factors are critical, as they influence the interpretation of cytotoxicity data and distinguish specific anticancer effects from general cytotoxicity or solvent-related toxicity [96]. Future studies should incorporate these controls and comparisons to enhance cytotoxicity evaluations’ translational relevance and robustness.

## 4. Conclusions

This study developed a novel freeze-dried fermented camel milk (FCM) enriched with Ajwa date pulp (ADP), enhancing its nutritional and functional properties, such as dispersibility and probiotic survivability, particularly for *L. acidophilus* and *B. bifidum*, supporting gut health applications. The product showed promising antidiabetic activity, though anticancer effects were more potent in non-ADP samples, highlighting a complex interaction between bioactive peptides and proteolysis. Interestingly, the study paves the way for innovative dairy-based interventions that align with global health priorities by integrating prebiotic, probiotic, and postbiotic components. The findings advocate for continued research to refine production processes and expand the applications of this promising functional food. Despite the benefits, limitations include the cost of freeze-drying and scalability challenges. Additionally, a key limitation of our study is the lack of comparative analyses using standard anticancer agents and comprehensive controls, which hampers contextualization of the potency and specificity of our compounds. Future studies should incorporate comprehensive and comparative analyses to enhance the reliability of cytotoxicity assessment while also optimizing processing and peptide fractionation to identify active compounds, conducting clinical trials to confirm health benefits, and exploring different date varieties and probiotic strains to advance functional dairy foods targeting noncommunicable diseases.

## Figures and Tables

**Table 1:** Reconstitution properties of FCM and FCM incorporating ADP fermented by either ATB-5 or ATB-5 with *L. rhamnosus* (mean ± SE) (*n* = 6).

Properties	FCM Incorporating ADP **					
	FCM	FCM+L	FCM12D	FCM12D+L	FCM15D	FCM15D+L
Density properties (g mL^−1^)						
Loose bulk density (LBD)	0.22 ± 0.00 ^d^	0.24 ± 0.01 ^c^	0.32 ± 0.01 ^b^	0.32 ± 0.01 ^b^	0.36 ± 0.01 ^a^	0.35 ± 0.01 ^a^
Packed bulk density (PBD)	1.24 ± 0.02 ^b^	1.26 ± 0.02 ^b^	1.41 ± 0.02 ^a^	1.41 ± 0.02 ^a^	1.47 ± 0.02 ^a^	1.44 ± 0.02 ^a^
Particle density (PD)	3.03 ± 0.06 ^a^	2.36 ± 0.05 ^b^	2.17 ± 0.05 ^c^	1.75 ± 0.04 ^d^	1.61 ± 0.03 ^e^	1.64 ± 0.03 ^d,e^
Density of powder solids	1.31 ± 0.01 ^b^	1.31 ± 0.01 ^b^	1.36 ± 0.01 ^a^	1.36 ± 0.01 ^a^	1.38 ± 0.01 ^a^	1.38 ± 0.01 ^a^
Flowability						
Carrier index (CI%)	82.13 ± 0.66 ^a^	80.63 ± 0.72 ^a^	77.24 ± 0.84 ^b^	77.33 ± 0.84 ^b^	75.17 ± 0.51 ^b^	75.50 ± 0.50 ^b^
Hausner ratio (HR)	5.61 ± 0.21 ^a^	5.18 ± 0.19 ^a^	4.41 ± 0.16 ^b^	4.42 ± 0.17 ^b^	4.03 ± 0.08 ^b^	4.09 ± 0.08 ^b^
Air content (mL 100 g^−1^ powder)						
Interstitial air content	47.62 ± 1.76 ^a^	37.12 ± 1.92 ^b^	24.84 ± 1.88 ^c^	14.05 ± 2.10 ^d^	5.88 ± 0.720 ^e^	8.13 ± 0.68 ^e^
Occluded air content	31.78 ± 0.70 ^e^	41.15 ± 0.90 ^d^	44.77 ± 0.98 ^c^	55.76 ± 1.21 ^b^	60.92 ± 1.32 ^a^	59.74 ± 1.30 ^a^
Porosity (%)	58.97 ± 1.38 ^a^	46.60 ± 1.80 ^b^	34.94 ± 2.19 ^c^	19.68 ± 2.70 ^d^	8.63 ± 1.080 ^e^	11.75 ± 1.02 ^e^

**: FCM, fermented camel milk with ABT-5 (0.7 g L^−1^); FCM+L, fermented camel milk with ABT-5 (0.35 g L^−1^) + 15 mL of *L. rhamnosus*; FCM12D, fermented camel milk with ABT-5 (0.7 g L^−1^) incorporating 12% ADP; FCM12D+L, fermented camel milk with ABT-5 (0.35 g L^−1^) incorporating 12% ADP + 15 mL of *L. rhamnosus*; FCM15D, fermented camel milk with ABT-5 (0.7 g L^−1^) incorporating 15% ADP; and FCM15D+L, fermented camel milk with ABT-5 (0.35 g L^−1^) incorporating 15% ADP + 15 mL of *L. rhamnosus* culture. ^a,b,c,d,e^: No significant difference (*p* > 0.05) exists between any two means within the same row with the same superscripted letters.

**Table 2:** Instrumental and visual color analysis of FCM and FCM incorporating ADP fermented by ATB-5 or ATB-5 with *L. rhamnosus* (mean ± SE) (*n* = 6).

Treatments ^#^	Instrumental Color Parameters						
	L*	a*	b*	C	H°	BI	∆E
FCM	87.94 ± 0.04 ^a^	−2.53 ± 0.10 ^b^	1.61 ± 0.08 ^c^	3.16 ± 0.17 ^c^	147.45 ± 0.26 ^a^	−0.43 ± 0.19 ^e^	0 ± 0 ^e^
FCM+L	88.16 ± 0.09 ^a^	−2.71 ± 0.19 ^b^	3.34 ± 0.16 ^b^	4.19 ± 0.14 ^b^	128.94 ± 0.68 ^b^	1.67 ± 0.20 ^d^	1.88 ± 0.18 ^d^
FCM12D	69.52 ± 0.09 ^b,c^	−0.69 ± 0.21 ^a^	16.45 ± 0.31 ^a^	16.47 ± 0.32 ^a^	92.34 ± 0.69 ^c^	25.60 ± 0.34 ^b,c^	23.75 ± 0.17 ^b,c^
FCM12D+L	70.50 ± 0.18 ^b^	−0.55 ± 0.13 ^a^	16.38 ± 0.15 ^a^	16.39 ± 0.15 ^a^	91.89 ± 0.45 ^c^	25.20 ± 0.38 ^c^	22.96 ± 0.22 ^c^
FCM15D	68.36 ± 1.23 ^c,d^	−0.54 ± 0.16 ^a^	17.10 ± 0.17 ^a^	17.11 ± 0.16 ^a^	88.21 ± 0.57 ^d^	27.51 ± 0.95 ^a,b^	25.08 ± 1.02 ^a,b^
FCM15D+L	67.61 ± 0.53 ^d^	−0.46 ± 0.05 ^a^	17.14 ± 0.52 ^a^	17.15 ± 0.52 ^a^	88.50 ± 0.14 ^d^	28.04 ± 1.21 ^a^	25.68 ± 0.73 ^a^

L*, a*, and b* were obtained directly from the Hunter instrument. C, H°, BI, and ∆E were calculated according to the formulated equations in the Methods section, Equations (1)–(4), based on their values in control FCM. ^#^: FCM, fermented camel milk with ABT-5 (0.7 g L^−1^); FCM+L, fermented camel milk with ABT-5 (0.35 g L^−1^) + 15 mL of *L. rhamnosus*; FCM12D, fermented camel milk with ABT-5 (0.7 g L^−1^) incorporating 12% ADP; FCM12D+L, fermented camel milk with ABT-5 (0.35 g L^−1^) incorporating 12% ADP + 15 mL of *L. rhamnosus*; FCM15D, fermented camel milk with ABT-5 (0.7 g L^−1^) incorporating 15% ADP; and FCM15D+L, fermented camel milk with ABT-5 (0.35 g L^−1^) incorporating 15% ADP + 15 mL of *L. rhamnosus* culture. ^a,b,c,d,e^: No significant difference (*p* > 0.05) exists between any two means within a column with the same superscripted letters.

**Table 3:** Inhibition of α-amylase and α-glucosidase and anticancer activity of probiotic fermented camel milk supplemented with ADP at 0, 12, and 15% (mean ± SE) (*n* = 6).

Treatments		IC_50_ (μg mL^−1^)	
	α-Amylase	α-Glucosidase	Anticancer Activity
FCM	8.37 ± 0.003 ^e^	10.74 ± 0.01 ^e^	82.22 ± 0.3 ^f^
FCM+L	40.47 ± 1.17 ^c^	27.90 ± 1.1 ^c^	85.19 ± 0.72 ^e^
FCM12D	14.83 ± 0.01 ^d^	17.49 ± 1.01 ^d^	94.89 ± 0.53 ^d^
FCM12D+L	47.80 ± 1.12 ^b^	47.36 ± 2.21 ^b^	293.24 ± 8.71 ^b^
FCM15D	42.31 ± 1.31 ^c^	46.44 ± 0.75 ^b^	275.51 ± 1.1 ^c^
FCM15D+L	111.43 ± 2.10 ^a^	77.21 ± 1.21 ^a^	312.95 ± 1.4 ^a^
Acarbose	3.12 ± 0.03 ^f^	7.52 ± 0.01 ^f^	---

^a,b,c,d,e,f^: No significant difference (*p* > 0.05) exists between any two means within a column with the same superscripted letters.

## Data Availability

The original contributions presented in this study are included in the article. Further inquiries can be directed to the corresponding author.
